# Power, recovery and doing something worthwhile: A thematic analysis of expert patient perspectives in psychiatry education

**DOI:** 10.1111/hex.13375

**Published:** 2022-01-25

**Authors:** Katie Ward, Miriam Stanyon, Karl Ryan, Subodh Dave

**Affiliations:** ^1^ Derbyshire Healthcare NHS Foundation Trust Derbyshire UK

**Keywords:** expert by experience, expert patient, medical education, mental health, public involvement, qualitative

## Abstract

**Background:**

Patient involvement in psychiatry education is required by policy and has many benefits for students. Little research has focused on the impact on expert patients (EPs).

**Objective:**

This study aimed to explore the impact of involvement in psychiatry education on mental health patients.

**Design:**

A qualitative descriptive study using semistructured interviews was conducted in a psychiatry teaching unit in the East Midlands, UK. A purposive sample of 20 EPs involved in teaching was interviewed about the social and psychological impacts of involvement. Transcripts were analysed thematically and a coding scheme was developed.

**Results:**

Five themes were identified: shaping the doctors of the future—something worthwhile, challenging assumptions about mental health, recovery and transformation, vulnerability and support and expertise and power.

**Conclusion:**

These EPs benefitted from their experience of teaching. Involvement in psychiatry teaching may require putting oneself in a vulnerable position, but a supportive and open faculty team may mitigate this challenge. The Expert Patient Programme was seen as a way of helping to reduce the power difference between patients and doctors in the future. There is a need to examine the language that we use to talk about patient involvement as this may have implications for this power dynamic. The context and mechanisms that lead to the benefits described by participants should be studied so that these benefits may be generalized to other contexts.

**Patient Contribution:**

An EP was involved in the planning and ethical approval application process of the project and the drafting and approval of this manuscript.

## 1 BACKGROUND

Since the 1980s, the National Health Service in the United Kingdom has required that patients and the public be involved in all aspects of healthcare. The General Medical Council's requirements for patient and public involvement (PPI) in undergraduate medical education^1^ state that ‘partnership’ with patients and the public should be explicit in all aspects of the curriculum. Consequently, the role of patients in medical education has changed over recent years from a passive resource for student learning towards an autonomous partner in the design and implementation of education programmes.^2^, ^3^ The subject of patient involvement (PI) in medical education is not limited to the United Kingdom, and work is being undertaken worldwide, especially in Canada, the United States and Australia.^4^, ^5^, ^6^ PI in psychiatry education is especially pertinent as there are elements of psychiatry teaching that have the potential to disempower and stigmatize mental health patients, such as deprivations of liberty and forced medication.

PI in medical education has taken many forms. The language surrounding PI is borrowed from the community activism movement of the 1960s,^7^ especially the concept of a ‘ladder of engagement’, where the public have increasing power over decisions concerning them. A taxonomy of PI in education categorizes the patient's role according to the level of involvement,^3^ ranging from case scenarios (Level 1), through to involvement at the institutional level in teaching, evaluation and curriculum development (Level 6). Participation at this level goes beyond partnership into coproduction, where expertise by training and by experience is valued equally in the development of teaching resources. It is coproduction that is increasingly being seen as the gold standard of healthcare professional education worldwide.^8^

The nomenclature in PI is varied. The terms ‘volunteer patient’,^2^, ^9^ ‘expert patient’, ‘client’,^10^ ‘expert by experience’^11^ and ‘service user’^12^ are all used and each discipline has its preferred terminology. The majority of participants in this study preferred the term ‘expert patient’ (EP) and it will be used throughout this article. The terms used to refer to EPs have connotations for how they are perceived by students, faculty members and themselves, and have implications for the power dynamics of teaching interactions.^11^ Theories such as Situated Learning^13^ and Dramaturgy^14^ have been used to explain changes in power balance that take place when a patient becomes a teacher.^15^, ^16^ Studies have shown that EP involvement in teaching can lead to a cultural shift where the power balance is improved and patient perspectives are valued.^12^, ^17^, ^18^, ^19^

Involvement of EPs in medical education benefits students by increasing learner satisfaction,^20^ improving communication skills,^21^ increasing empathy and improving understanding of the patient's perspective and patient‐centred care.^20^, ^22^, ^23^, ^24^, ^25^ Yet, it is vital to evaluate the impact of PI on the EPs involved.^26^ Previous studies have found benefits for EPs such as better knowledge of their own health problems,^21^, ^27^, ^28^, ^29^ a deeper understanding of health services and doctor–patient relationships,^21^, ^29^ personal satisfaction,^30^ empowerment and increased confidence^24^, ^27^, ^28^, ^29^, ^31^ and developing a comprehensive narrative.^21^, ^29^ However, much of this study has been carried out in general medical education. The impact of involvement on mental health patients may be different to that of patients in other fields. In the field of mental health nursing, guidelines on the involvement of EPs in teaching have been coproduced,^8^ and research has explored how EPs perceive their role and their impact on students.^32^, ^33^, ^34^ EPs report feeling empowered by their involvement in teaching, and one similar study in the context of pharmacy education has shown that involvement in teaching can lead to feelings of empowerment in mental health patients, can help to develop a recovery narrative and to re‐evaluate the maintenance of their condition,^31^ but the impact of involvement on mental health patients has not been fully explored. There have been concerns that mental health patients are more vulnerable to distress and the exacerbation of their illness through teaching.^29^, ^35^ Therefore, the aim of this study is to explore the impact of involvement in psychiatry teaching on EPs.

## 2 METHODS

### 2.1 Theoretical position and study design

This study adopted a qualitative descriptive design^36^ using face‐to‐face semistructured interviews with EPs to identify the impact of teaching involvement on EPs. This study adopted a contextualist critical realist perspective.^37^ Critical realism as originally espoused by Bhaskar^38^ sees reality as layered and seeks to explore causative mechanisms for what is experienced and observed. In this way, it illuminates the complexity of healthcare, though recognizing that knowledge of this complexity is filtered through an interpretive lens.^39^

### 2.2 Setting and sample

This study was carried out at a psychiatry teaching unit (PTU) in the East Midlands, which has operated an Expert Patient Programme (EPP) for teaching medical undergraduate psychiatry for 12 years. Mental health patients are recruited to the EPP through word of mouth, leaflets in GP settings and referral by consultants. There are eight cohorts of 15 students attending their 5‐week psychiatry placements per year. In history‐taking practice, EPs allow students to practice history‐taking in one‐to‐one interviews and feedback to students on how they felt during the encounter and the students’ demonstration of communication skills, empathy and professionalism. In ‘Key case’ sessions, teaching about symptoms, diagnosis and treatment is interwoven with the EPs' recovery narrative to allow students to see the patient perspective. In ‘Masterclass’ sessions, students are given the opportunity to assess an EP as if in clinic and to discuss with the EP the context of their condition and how it affects the EP in day‐to‐day life. Throughout all activities, EPs teach students about the patient perspective and the importance of quality of life and recovery. There is an ethos of collaboration between faculty members and the EPs in facilitating all sessions, valuing experiential and professional expertise equally. EPs are supported through preparatory emails, a ‘check‐in’ immediately before the session, a faculty member present throughout and the opportunity for a telephone de‐brief afterwards. Forty EPs currently participate in teaching at the PTU. Two lived experience educators are employed as faculty members. EPs are remunerated for their teaching (£15 per session), and all expenses are reimbursed.

All 40 EPs were invited to participate. Twenty EPs were willing to be interviewed. Recruitment ceased after 20 interviews as no new themes were arising and data saturation was thought to have been reached.^40^

### 2.3 Data collection

An interview guide was developed with the purpose of eliciting EP narratives about their involvement in teaching. The guide was piloted, and changes were made to clarify meaning and encourage a fuller response. Interview guide questions were based on themes originating from a review of the literature on PI in teaching. Topics in the interview guide included motivations for joining the EPP and continued participation, support received, experience of the EPP, advantages and challenges to participation, understanding of recovery and thoughts about what it means to be an EP. Open questions were supplemented by prompts to facilitate exploration of themes. Interviews were conducted in a private room at the PTU between October 2018 and July 2019. No demographic information was collected from participants to maintain anonymity. Each interview lasted approximately 1 h. Interviews were audio‐recorded and anonymized on transcription. Interviews were conducted by the first author, a female qualitative sociologist and psychiatrist, with a background in health‐related research. The first author had no involvement in the EPP before the interviews.

### 2.4 Data analysis

An inductive thematic analysis process^37^ was used to analyse the sections of the data set pertaining to the EPs’ experience and reflections upon involvement in the EPP. When identifying themes, the first author adopted a semantic approach, where she sought to describe, summarize and interpret data. The analysis followed the following six phases, with the second author becoming involved in Stages 4–6: (1) careful reading of transcriptions by the researcher to become familiar with the content and potential areas of interest, (2) generation of initial, provisional codes, (3) searching for themes and grouping codes into themes, (4) reviewing of themes across cases and final coding of material, (5) defining and naming of themes, identifying major areas of interest and alternative viewpoints held by individual patients, and (6) producing a report that accurately represents these themes through the selection of illustrative examples.^37^

### 2.5 Ethical considerations

Ethical approval was granted by the East Midlands Research Ethics Committee. Participants understood that the participant's care team may be contacted if there was any distress. If the participant wished, their care coordinator was informed of his or her participation in the project.

## 3 RESULTS

### 3.1 Findings

#### 3.1.1 Theme 1: Shaping the doctors of the future—something worthwhile

The majority of participants stated that their original motivation for involvement in the EPP was to ‘give something back’ to services that had supported them in their illness.

*They've helped me over a period of about 20 years with my mental health. Some of them I still know now, so it's nice to come here and give something back and help*. (Participant 4)

However, after involvement had commenced, this motivation evolved into the realization that they were offering a unique contribution.

*I just love the fact that I get to help them…someone helps me, I help other people. I'm actually helping people who are going into the medical profession have a clearer understanding of how to help other patients*. (Participant 14)

Many of the EPs spoke of the sense of well‐being gained from knowing that they were contributing to medical teaching, improving healthcare for others. This contribution was felt to be ‘worthwhile’, leading to increased self‐confidence, compared to other aspects of life that made them feel ‘purposeless’, such as unemployment.

*I do need to feel worthwhile and I think that it is one of those things that makes me feel a little bit more worthwhile…when doing a lunch time seminar and it's a group of students, particularly if there's a good interaction and they're asking lots of questions I think,‘yeah, I do enjoy those*’. (Participant 4)

Contributing to medical education was seen as a way in which they can effect real change at the source, by shaping the doctors of the future.

*It's all well and good helping the actual person who needs the care, but if you're not helping change the way people are providing the care, then they are continually going to cycle back through the system*. (Participant 14)

Most participants appreciated that the experience of mental illness was something that students could not learn through other means of study. They discussed the importance of students learning directly from patients to increase their awareness of the issues experienced by patients and thereby to improve empathy and understanding.

*It's like training a mechanic without giving them a car to fix…speak to somebody that's scared to go to Asda. Speak to somebody that punches themselves in the face… yes read the books, but speak to service users that are prepared to give them themselves… It's lived*. (Participant 2)

#### 3.1.2 Theme 2: Challenging assumptions about mental health

Many participants spoke about mental health stigma as a long‐standing concern for people with mental health problems and how it can be restrictive, leaving them feeling under‐valued and an outsider to wider society. Many participants considered involvement in the EPP an opportunity to find their own voice and ‘speak back’ to those who had previously denigrated them. The following participant talks about her experience of having a diagnosis of borderline personality disorder, the perceptions she has encountered and her desire to change those views:

*I think borderline is still not super well‐known and there's a lot of stigma about them being really difficult…hopefully I can be a nice human face to it. If someone comes in and is borderline, instead of thinking some sort of awful stereotype, then [the medical students] think about something that I've said and it'll help them, ‘oh ok, this is a person and they've got a story as well’* (Participant 3)

Finding the conviction to ‘speak up’ about their own mental ill health was a form of activism for many EPs. Making their own experiences public and working for better recognition and treatment validated their experiences and led to increased personal value and self‐esteem. These positive feelings were deepened by the reactions and feedback received from medical students.

*When the student says ‘thank you for sharing your story, I didn't realise that that kind of world exists’—opening their eyes and acknowledging that you've helped them shift their thought process, that is the best thing…They do say it's a privilege a lot of them…even now as I'm saying it I can feel tears at the back of my eyes*. (Participant 5)

#### 3.1.3 Theme 3: Recovery and transformation

One of the reported gains of participating in the EPP was the opportunity to construct a recovery narrative. This gave them a sense of creating order out of chaos, that things were progressing and that there was a positive future to look forward to. Many participants spoke of the way in which the repetition of their story to successive cohorts of students was therapeutic and helped to consolidate feelings about past traumatic events.

*The more you repeat it the more you accept that it's happened. Once you accept that things have happened to you and realise that there is nothing that you can do to change it, and that it is what it is, but you can still progress forward, that is when you can move forward* (Participant 14)

One participant spoke of the way in which creating a recovery narrative and seeing her experiences as a coherent whole helped her to recover supressed memories and removed some of the traumatic emotional elements.

*I realise, for example, that I've closed off a lot of memories that I've found difficult. I mean this is stemming from childhood. So each time I'm asked to give some information I remember a little bit more and that helps… and it's easier to make it more matter‐of‐fact rather than huge emotional trauma*. (Participant 5)

The following participant commented that contributing to the EPP gave them the opportunity to construct an organized, linear narrative.

*When I first came, I was a bit jumbled up but now, I've sort of put it into perspective and it's got a beginning, a middle and an end… It clears your head*. (Participant 8)

Some participants spoke of the way in which involvement in the EPP helped them to see the future steps in their recovery. The following participant viewed her involvement in the EPP as an opportunity to maintain her mental health and to keep challenging herself as a means of recovery:

*Before, a few years ago I was so scared of everything, I mean I wouldn't talk to people…keeping doing things that make me anxious is quite important cos I've got to keep pushing myself otherwise it comes back and everything shrinks again*. (Participant 3)

Taking up a formal position as an EP was an important step for most EPs and some likened it to a ‘stepping stone’ back into social contact and/or employment. Some EPs expressed a desire to work in the area of mental health, emphasizing the extent to which their own experiences of mental ill health and recovery could help others and influence the way in which services are delivered.

*I'm hoping, at some point, I can get a part time job, paid, within the mental health community. I would like to work with patients… I'm on benefits and I've never been on benefits in 37 years…it's a rung on a ladder*. (Participant 2)

Closely linked to the concept of recovery was that of transformation. Most EPs discussed the way in which the EPP took distressing and negative experiences and transformed them into something they perceived as worthwhile and valuable to wider society.

*I can now turn all of that negative stuff into something that will help future patients because the doctors are better prepared, that's huge!* (Participant 5)

Some participants also spoke of the sense of privilege that they felt in teaching, which not only increased their own self‐esteem but also gave hope that society could view experiencing mental illness as an asset.

*In a way I'm honoured that I do this because it's a special thing, to be able to educate… So this is amazing what they're doing, that they'd want to take my learning experiences of having mental health*. (Participant 8)

#### 3.1.4 Theme 4: Vulnerability and support

The learning activities involve EPs telling their story of mental illness or having their history of mental illness or traumatic events taken, and some participants said that this can be upsetting and makes them vulnerable to exacerbation of their condition.

*I do get anxious. The main thing I get anxious about is that something might trigger something, because obviously they don't know me well enough*. (Participant 2)

It was also acknowledged that there are times when participation would be detrimental as talking about one's own mental health was likely to compound personal problems. Knowing when to take a break from the EPP was important and an indication of valuable self‐knowledge:

*If you were in that kind of place, then talking about the whole history of your entire illness—that would keep you in that trauma*. (Participant 3)

EPs talked about the robust system of support provided by faculty members at the PTU. This was important for many EPs and suggested that the support structure is a major factor that helps maintain EP involvement.

Some EPs spoke of the way in which faculty members are alert to the possibility of EPs experiencing personal difficulties during the re‐telling of a personal history and how measures are in place to attempt to mitigate any potential harm. One participant gave an example of the thorough and discreet support received on a specific occasion:

*After the session had finished [faculty member] could see, that I was struggling…he stayed in and had a chat with me and I ended up crying and he wanted to make sure I was safe, that I wasn't going to get home and do something silly. We talked a bit and he was able to give me some comfort…Then he talked about getting in touch with my CPN… So I agreed that he could tell my CPN. But he did it very gently, very supportive*. (Participant 5)

Support for EPs was also described as the general ethos of the EPP. Participants were aware that support did not just revolve around the teaching activities themselves but that there was an underlying philosophy of care for the EPs, generating trust and respect.

*It's shown that we're valued by the team here and I think that's really important…you can always come in and they're like ‘sit down and have a cup of tea’.…it's not just come here, dredge up all of all your sorrows and then, ‘see ya'!’* (Participant 3)

This participant also spoke of the culture of openness about faculty members' own mental health. This was thought to remove some of the perceived taboos of talking about mental health issues.

*‘Cos they're quite open about their own mental health—there's a good openness thing. I know in a lot of the world it is still a bit taboo* (Participant 3)

#### 3.1.5 Theme 5: Expertise and power

The EP's thoughts and feelings around the terminology used to describe their role were emotive and showed how the EPP disrupts traditional power relations between doctors and patients. Participants would sometimes refer to the power imbalance that they had experienced between doctors and patients and how this power often revolved around who had the knowledge or expertise in mental health.

*A lot of the time you go in and the doctor's there and ‘high and mighty’…and because they have all the knowledge it feels like they have all the power because they're the ones saying this is what's wrong with you. But in actual fact you can also know that there's something wrong… they should respect you and the fact that you're saying something*. (Participant 14)

Referring to patients as ‘experts’ and allowing them to play a key role in the way in which medical education is delivered appear to represent, for the participants, the shifting of power and control from doctors to patients.

*There's no hierarchy, which, with mental health you often get…They do respect you and, as [faculty member] says, we are the experts, so in a way we're in control…So I think the equality is quite good, I don't feel intimidated or anything*. (Participant 7)

Another participant spoke of the way in which they had to justify their right to be recognized as an expert to their friends, and to themselves, and that being an EP was not an attempt to elevate themselves above their station.

*My friends are automatically like…‘Oh okay, so you're basically a loony in doctor's clothing…you're trying to be on the same level as them’…They believe what I believed previously: doctors are here [points up], patients are here [points down]*. (Participant 14)

Some participants wanted to distance themselves from the term ‘expert patients’, saying that it represented a contradiction in terms of knowledge, power and responsibility.

*If I'm an expert photographer… I take lots of photographs, and they're very good quality. That's an expert. An ‘expert patient’, a patient who lies in bed and gets seen to by doctors, that seems to me like, ‘I'm great at being ill’*. (Participant 5)

For a minority of participants, ‘expert by experience’ was a preferred term to recognize a different type of expertise derived from what they had lived through:

*I like ‘expert by experience*’*… Because it's what I am—I am an expert in my mental illness and I'm an expert on what I've been through in my life so I'm an expert by experience. I'm not an expert with full qualifications*. (Participant 5)

## 4 DISCUSSION

This is one of the few studies that explores the impact of involvement in psychiatry teaching exclusively on the patients involved. The findings in this study are broadly consistent with those of other research in psychiatry education, such as the sense of empowerment resulting from the belief in making a difference to students, the sense of satisfaction in reducing mental health stigma and the way in which involvement in teaching allows EPs to gauge and maintain their own recovery.^31^ Yet, this study has also found that engagement in teaching can not only help EPs in maintaining their recovery but it can also be seen as a stepping‐stone to further recovery and can help them transform their negative experiences into something positive. This study has also examined the concept of vulnerability in more detail and explored the way in which faculty support is key to mitigating against the emotion and distress that can be triggered through involvement.

Consistent with previous studies, EPs were initially motivated by wanting to ‘give something back’^3^, ^41^ and contribute to medical training^9^, ^42^; however, this study found that patients were also aware of the personal benefits of involvement. The decision to take part was never passive, but taken autonomously, weighing the costs and benefits of involvement. Involvement continued because they appreciated the importance of their unique contribution to the curriculum and the resulting increase in their self‐esteem. Other forms of PI in teaching, such as simulated or standardized patients, do not involve the opportunity to share unique, and diverse, experiences. It is possible that the same win–win dynamics of involvement would not be experienced by those involved in this manner and it is therefore important that different categories of involvement are defined and demarcated in research studies on this topic.

Participants believed in the importance of medical students learning from mental health patients. Although medical students are exposed to mental health patients through their clinical placements, the EPs felt that the EPP contributes over and above that clinical experience. Theories of power dynamics in teaching interactions indicate that the difference may be related to the setting of teaching encounters. According to Goffman's dramaturgy,^14^, ^15^ the patient in the clinical setting functions as a passive ‘prop’, whose role is characterized by passivity and illness, whereas in the academic setting, their role is characterized by expertise and recovery. The patients in our study appreciated the value of students’ exposure to mental health patients in a position of power and influence rather than in the traditional patient role, something also appreciated in the mental health nursing literature,^43^ and made a logical connection to students’ treatment of patients in the future.

The EPs always portrayed a balanced power dynamic between the EPs, students and faculty members during teaching. EPs spoke of feeling valued and perceived as experts by faculty members and students. This contrasts with other literature where PI programmes have found the power dynamics difficult to navigate, making true collaboration challenging.^44^ It is therefore important, according to a critical realist perspective, to examine the context of and mechanisms by which this power balance was achieved.^45^ The authors would argue that the support that EPs received from faculty members and the openness with which they spoke about their own mental health reduced the us/them mentality often existing between patients and faculty members. Employment of two lived experience educators demonstrates the value placed on lived experience by this PTU. It is also interesting that, although EPs in this study were involved only to Level 4 of Towle's taxonomy of participation,^33^ there seemed to be no indication that they desired any greater level of involvement or control. This may be because they were unaware of the possibility of a greater level of involvement. Yet, whenever a theoretical perspective borrows rhetoric from another movement, such as community activism, one must remain critical of the extent of its applicability.^21^ We would argue for judiciously establishing the purposes of PI in education and ensuring that PI meets those purposes. This is not an argument to stop fighting for coproduction and PI at the highest levels of medical education, but to determine the level of involvement and the contextual factors that create the most positive impact for EPs, students and faculty members. This will prevent the extremes of EPs feeling like ‘a live body to be poked and prodded’^22^ or faculty members being afraid of exerting any power, despite their expertise.^44^ Guidelines such as those produced in the mental health nursing context^8^ could be helpful in establishing effective coproduction in the area of psychiatry education.

Some EPs disliked the terminology of ‘expert patient’. Previous literature has outlined the connotations of using certain language to describe EPs.^27^, ^28^ Similar to the term ‘service user’ and its association with drug addiction,^11^ the literature suggests that using the term ‘patient’ will immediately mobilize the patient–doctor relationship power dynamic and therefore disempower the EP in the educational setting.^13^ The reason a minority of participants disliked the term ‘expert patient’ was not because they felt disempowered in relation to faculty members, but because of the association with an expertise in being ill. It may be that, in academia's quest to be politically correct and careful not to disempower through our labelling, we fail to realize that the general public hold other associations with certain labels. In mental health nursing, the term ‘expert by experience’ is predominantly used,^32^, ^46^ though the terms ‘expert patient’ and ‘patient educator’ are more commonly used in the field of medicine. The authors of this paper chose to honour the majority decision of the participants in how they wished to be described, yet also give voice to the minority. The arguments raised by these participants in this study illustrate that there is never one coherent ‘patient voice’^7^ and researchers should not only report on the views of the majority.

This study sought to explore the impact on mental health patients in particular. It is interesting to note that, for this group of mental health patients, benefits such as increased self‐esteem and self‐confidence, empowerment and development of a comprehensive narrative appeared to be experienced on a more profound level than for other patient groups. Although developing a narrative had been seen as a benefit in other patient groups,^21^, ^29^ this appeared to be experienced to a deeper level, and for some, even led to recovery of previously supressed memories. The increase in confidence and self‐esteem was even more impactful for those who struggle with these as part of their mental health condition. The positive impact is not inconsequential when we see that EPs view involvement in the EPP as a strategy for maintaining their current mental health and a stepping‐stone for re‐entering employment. Although it is not feasible or appropriate for all mental health patients to get involved with a psychiatry teaching programme, the underlying mechanisms for the improvements reported in EPs’ mental health should be established so that the same therapeutic benefits may be gained from other interventions.

In accordance with previous literature, the participants of this study stated that recounting their history could be distressing and could exacerbate their condition.^29^ There was a need for faculty members and EPs to know when an EP was not in the ‘right place’ to take part in teaching activities, and the interviews show that support from faculty members was key to the EPs’ willingness to be vulnerable in sharing their histories. Again, this may be even more crucial in the area of mental health, where many patients may never see themselves as fully recovered but only as constantly maintaining a level of mental wellness.

### 4.1 Strengths and limitations

This study sought to explore the impact of involvement in psychiatry education on mental health patients and is adding to a corpus where there is currently a dearth of literature. The qualitative approach meant that internal perspectives, which would have been lost using quantitative methodologies, were revealed, for example, the reason EPs do not like the term ‘expert patient’, a level of distress that EPs may be willing to undergo for the good of the students and the PTU.

As with any qualitative work, the views expressed are the views of these participants only, and they spring from the experience of teaching in this context. We must always ‘pay attention to the silences’^26^ and seek to make sense of ambiguities and absences in the data. The authors of this study chose not to collect demographic data to maintain anonymity and this does limit the readers’ ability to draw conclusions about which voices are missing. Despite limitations of transferability, this study presents the context from which reported benefits flow for example a robust support system, an ethos of openness about mental health, faculty members with lived experience and the value and respect bestowed on EPs by students. The authors argue that the reported benefits may be transferable to other contexts with these characteristics.

### 4.2 Future directions

Future research should seek to critically examine the reasoning behind PI in medical education, scrutinizing power dynamics to ensure that PI benefits all. The community activism movement would assume that patient control is the gold standard of PI, yet coproduction would seem to be a more effective aim, though currently there is no evidence to confirm this. Even if this is not the focus of studies, authors should make their theoretical assumptions about PI explicit in their articles. Developing standards of coproduction in the area of psychiatry, similar to those developed in other disciplines, could contribute to the effectiveness of these working relationships.

The therapeutic nature of involvement in psychiatry education for mental health patients should be examined further. The benefits to EPs described here show that EPs could be experiencing clinical benefits as a result of involvement. Future work should build a theoretical model for these benefits and seek to examine these empirically.

## 5 CONCLUSION

Participants in this study benefitted from their experience of teaching as they felt that they were involved in something ‘worthwhile’ and making a unique contribution to the education of doctors, helping to change negative assumptions about mental illness, developing a recovery narrative and seeing their negative experiences transformed into something positive. Although it was acknowledged that this often required putting themselves in a vulnerable position, the support and openness of the faculty team enabled participants to do this from a place of feeling safe and valued. Participants were aware of the power dynamics operating between the patient and the doctor and saw the EPP as a way of helping to reduce this power difference in the future. The EPs' views about the term ‘expert patient’ also expose the need for an agreed nomenclature in the area of PPI and one that accounts for the public's assumptions connected to certain terms. The EPs in this study experienced benefits from involvement in psychiatry education, and the context and mechanisms that produced these should be studied so that they may be transferred to other contexts.

## CONFLICT OF INTERESTS

The authors declare that there are no conflict of interests.

## Data Availability

The transcripts for the interviews of this study are available upon request.
